# Associations between appetite loss and psychopathology as well as gender differences in adolescents with major depressive disorders

**DOI:** 10.1186/s12888-025-07718-y

**Published:** 2025-12-25

**Authors:** Jinyue Xue, Lewei Liu, Jingwen Shang, Yachen Feng, Xianlin Sun, Jiawei Wang, Changhao Chen, Zhiwei Liu, Feng Geng, Daming Mo, Xiangfen Luo, Xiangwang Wen, Lei Xia, Huanzhong Liu

**Affiliations:** 1 Department of Psychiatry, The Fourth Affiliated Hospital of Anhui Medical University, 64 Chaohu North Road, Hefei, Anhui Province 238000 China; 2 Department of Psychiatry, School of Mental Health and Psychological Sciences, Anhui Medical University, Hefei, Anhui Province 230032 China; 3 Department of Psychiatry, Bozhou People’s Hospital, Bozhou, Anhui 236800 China; 4 Department of Psychiatry, Suzhou Second People’s Hospital, Suzhou, Anhui Province 234099 China; 5 Department of Psychiatry, Fuyang Third People’s Hospital, Fuyang, Anhui Province 236044 China; 6 Department of Psychology and Sleep Medicine, The Second Affiliated Hospital of Anhui Medical University, Hefei, Anhui Province 230000 China; 7 Department of Psychiatry, Anhui Province Second People’s Hospital, Hefei, Anhui Province 230041 China; 8 Department of Psychiatry, Ma’anshan Fourth People’s Hospital, Ma’anshan, Anhui Province 243031 China

**Keywords:** Appetite loss, Psychopathology, Gender differences, Adolescents, Major depressive disorder

## Abstract

**Background:**

Appetite loss is frequent in patients with major depressive disorders (MDD). However, research on the association between appetite loss and psychopathology symptoms in adolescents with MDD remains limited, particularly regarding gender differences. Therefore, this study aimed to explore these associations and potential gender differences.

**Methods:**

A cross-sectional study carried out between January to December 2021 in eight hospitals, recruiting 364 adolescents with MDD. It utilized the Hamilton Depression Scale (HAMD), the Positive and Negative Suicide Ideation Scale for Adolescents (PANSI), the Chinese version of the Epworth Sleepiness Scale (ESS), the Insomnia Severity Index (ISI), and the Chinese version of the Internet Addiction Test (IAT) to assess patients’ depressive severity, suicidal ideation, sleepiness, insomnia, and internet addiction, respectively. Logistic regression was employed to examine the independent factors with an impact on appetite loss.

**Results:**

The prevalence of appetite loss was 69.2% in adolescents with MDD and 74.7% in female patients. Regression results indicated that appetite loss was independently correlated with Gender (OR = 0.559, 95% CI = 0.331 ~ 0.944, *p* = 0.029), BMI (OR = 0.925, 95% CI = 0.876 ~ 0.977, *p* = 0.005), HAMD score (OR = 1.080, 95% CI = 1.041 ~ 1.120, *p* < 0.001) and ISI score (OR = 1.073, 95% CI = 1.024 ~ 1.124, *p* = 0.003). When discussing gender differences, it was found that female patients were independently associated with BMI, HAMD score and ISI score, while male patients were only independently associated with HAMD score. Finally, the receiver operating characteristic (ROC) curve results showed that combining Gender, BMI, HAMD score, and ISI score predicted appetite loss well (AUC = 0.746). In female patients, the combined prediction of BMI, HAMD score, ESS score, and ISI score demonstrated predictive value for appetite loss (AUC = 0.732). In male patients, the HAMD score served as an effective predictor (AUC = 0.713).

**Conclusion:**

Adolescents with MDD, particularly females, faced a heightened risk of appetite loss. Additionally, appetite loss was significantly associated with multiple clinical symptoms, and these associations showed certain gender differences.

**Clinical trial number:**

Not applicable.

## Introduction

Major depressive disorder (MDD) is a frequent and serious mental disease in adolescents [1]. A Chinese epidemiological study showed that the prevalence of MDD in adolescents has reached 2.0% [2]. MDD not only affected the emotional state of adolescents, but was often accompanied by a range of somatic symptoms, in which changes in appetite are more common [3]. Appetite loss was frequently associated with depression, and a previous study showed that approximately half of patients with MDD experienced appetite loss [4]. Adolescents are still developing physically and mentally, and patients with MDD during this stage are more prone to eating disorders (ED) due to impaired emotions, cognition, and self-control [5]. However, despite previous studies investigating appetite loss in patients with MDD, research specifically targeting adolescents with MDD remains scarce. Given that appetite loss may impact treatment adherence and overall recovery, its prevalence and associated factors in adolescents with MDD warrant further investigation.

Research has indicated that appetite loss was strongly linked to a variety of psychopathological symptoms. For example, research has found that when adolescents experienced depressive moods, their eating behaviors were also influenced by these moods [6]. Another investigation showed that patients with appetite loss usually manifested more pronounced symptoms of depression [7]. There was also study that have found a correlation between appetite loss and suicidal ideation (SI) [8]. Additionally, studies have shown that excessive internet use may increase the risk of developing anorexia nervosa by causing adolescents to develop distorted body image [9, 10]. Notably, most of the aforementioned studies have focused on adults or individuals with anorexia nervosa, while research on adolescents with MDD remains insufficient.

Importantly, gender differences in these associations may exist, potentially arising from a multifaceted interplay of physiological, psychological, and sociocultural factors. For example, a 4-year longitudinal investigation revealed that female adolescents with MDD were more prone to developing ED than males, driven by factors such as body dissatisfaction, depressive symptoms, body mass index (BMI), and perfectionism [11]. Another clinical study further revealed that depression tends to be more severe when BMI was at lower levels in patients with chronic anorexia nervosa [12]. Females’ risk of depression appears to be more strongly influenced by changes in BMI. They often exhibit heightened concerns about their self-image and frequently resort to extreme dietary control to manage their weight, which not only exacerbates their psychological burden but also increases the risk of developing anorexia nervosa in the future [13, 14]. In addition, insomnia in females was also associated with appetite loss. A community-based study showed that females reported more appetite loss than males due to poor sleep [15]. Nonetheless, research on gender differences in the associations between appetite loss and a variety of psychopathological symptoms in adolescents with MDD remains relatively limited and warrants further in-depth exploration.

The above studies have shown that appetite loss may be linked with various factors, and that these factors differ across genders. However, existing studies have not systematically explored the potential mechanisms underlying these connections in adolescents with MDD. In particular, possible gender differences have been largely overlooked. Therefore, the study sought to investigate the associations between appetite loss and psychopathology in this population, including depressive severity, SI, sleepiness, insomnia, and Internet addiction (IA), as well as gender differences in these associations.

## Methods

### Participants

This study employed a multi-site, convenience-consecutive sampling method from January to December 2021, selecting adolescent patients with MDD from outpatient and inpatient departments across eight hospitals covering northern, central, and southern Anhui Province (the Fourth Affiliated Hospital of Anhui Medical University, the Fourth People’s Hospital of Hefei, the Second Affiliated Hospital of Anhui Medical University, Bozhou People’s Hospital, Suzhou Second People’s Hospital, Second Affiliated Hospital of Bengbu Medical University, Ma’anshan Fourth People’s Hospital, and Fuyang Third People’s Hospital). This approach ensured sample representativeness and minimized regional bias. All Participants were enrolled by qualified, board-certified psychiatrists or higher-level mental health specialists using DSM-5 criteria and standardized assessment tools. Evaluators from eight centers underwent centralized training, and all treatments were administered according to clinical guidelines. Inclusion criteria: (1) aged 12 to 18 years old, with adequate reading comprehension ability; (2) meeting the diagnostic criteria for MDD as defined in the Diagnostic and Statistical Manual of Mental Disorders, fifth edition (DSM-5), and independently diagnosed by two psychiatrists with the title of attending physician or above; (3) After obtaining written informed consent from the parent or legal guardian, and confirming through one-on-one communication that the subject fully understands the process, the subject voluntarily signs the informed consent form. Exclusion Criteria: (1) Diagnosed with schizophrenia, bipolar disorder, intellectual disability, or other mental disorders according to DSM-5; (2) Presence of severe infection, organic brain disease, autoimmune disease, or other major physical illnesses (e.g., gastrointestinal disorders, endocrine abnormalities or chronic diseases); (3) Use within the past 4 weeks of medications that may affect appetite, including but not limited to prokinetic agents, appetite suppressants, or appetite stimulants. In this study, the sample size was initially calculated using the formula n = Z²p(1-p)/d². Here, n represents the sample size, Z is the value corresponding to the 95% confidence interval (which was 1.96), d denotes the marginal error (which was set at 0.05), and p is the prevalence (Based on previous literature, approximately 65% of patients with MDD experienced appetite loss. Therefore, we set the p-value to 65% [16]). The minimum sample size obtained was 350. Finally, this study recruited 364 adolescents with MDD. The average age of all patients was 15.25 ± 1.63 years, their age at onset was 13.75 ± 3.64 years, duration of illness was 20.68 ± 16.91 months, and 70.6% of the patients were female (Table 1).

**Table 1:** Comparison of sociodemographic and psychopathological data of patients in the appetite loss and non-appetite loss groups in the total sample

Variables	Total sample (*N* = 364)	Appetite loss (*N* = 252)	Non-appetite loss (*N* = 112)	t/Z/χ^2^	*p*	Cohen’s d
Age (years), mean (SD)	15.25 ± 1.63	15.19 ± 1.64	15.40 ± 1.60	1.166	0.244	0.132
Gender, n (%)						
Males	107 (29.40)	60 (23.81)	47 (41.96)	12.314	0.001*	0.132
Females	257 (70.60)	192 (76.19)	65 (58.04)			
BMI (kg/m^2^), mean (SD)	21.22 ± 4.40	20.80 ± 4.38	22.17 ± 4.31	2.759	**0.006**	0.313
Age at onset (years), mean (SD)	13.75 ± 3.64	13.68 ± 4.14	13.91 ± 2.12	0.552	0.581	0.063
Duration of illness (moths), median (P_25_, P_75_)	15.00(8.00,27.00)	18.00(12.00,31.50)	12.00(6.00,24.00)	1.372 ^a^	**0.020**	0.072
Antidepressants, n (%)						
None	132(36.26)	95(37.70)	37(33.04)	2.004	0.367	
SSRIs	203(55.77)	140(55.56)	63(56.25)			0.074
Others	29(7.97)	17(6.75)	12(10.71)			
Study pressure, n(%)						
Small	20(5.49)	14 (5.56)	6(5.36)	3.224	0.119	
Average	141(38.74)	90 (35.71)	51(45.54)			0.094
Large	203(55.77)	148 (58.73)	55(49.11)			
HAMD score, mean (SD)	26.84 ± 7.68	28.46 ± 7.02	23.18 ± 7.86	-6.387	< 0.001*	0.725
PANSI score, mean (SD)	47.91 ± 11.90	49.61 ± 11.07	44.09 ± 12.84	-4.178	< 0.001*	0.474
ESS score, mean (SD)	9.49 ± 4.58	9.25 ± 4.62	10.04 ± 4.44	1.540	0.124	0.175
ISI score, mean (SD)	12.45 ± 5.87	13.48 ± 5.47	10.12 ± 6.08	-5.021	< 0.001*	0.594
IAT score, mean (SD)	51.68 ± 15.62	52.30 ± 16.01	50.30 ± 14.68	-1.124	0.262	0.128

BMI, body mass index; SSRIs, selective serotonin reuptake inhibitors; HAMD, Hamilton depression scale; PANSI, positive and negative suicide ideation inventory scale; ESS, Epworth sleepiness scale; ISI, insomnia severity index scale; IAT, internet addiction test

Bolded *p* value: < 0.05; SD: standard deviation

^a^ Mann-Whitney U-test. Cohen’s d indicates the effect size

* indicated variables with *p* < 0.05 after FDR adjustment

This study was approved by the Ethics Committee of the Fourth Affiliated Hospital of Anhui Medical University (202009-kyxm-04). Furthermore, all procedures conformed to the ethical standards of the 2013 version of the Helsinki Declaration (https://www.wma.net/policies-post/wma-declaration-of-helsinki/). Written informed consent was obtained from all participants and their legal guardians after they were fully informed about the study.

### Study instruments

#### Sociodemographic information

We collected detailed sociodemographic information from study participants by distributing self-administered questionnaires, including age, gender, BMI, academic stress, age at onset, duration of illness, and use of antidepressant medication.

#### Depressive

The Hamilton Depression Scale (HAMD) was used to assess the degree of patients’ depressive symptoms over the past week [17]. The scale includes 24 items and uses a 5-point rating system, and it has higher total scores being associated with greater severity of depressive symptoms. And HAMD has been widely used in studies of adolescents with MDD [18]. Based on previous research, the present study categorized patients according to their HAMD item 12 (gastrointestinal symptoms) scores. Participants scoring 0 points were defined as the non-appetite loss group (*n* = 112), while those scoring 1 or 2 points were defined as the appetite loss group (*n* = 252) [16]. To avoid potential confounding effects of this item, Item 12 was excluded when analyzing the correlation between depressive symptoms (assessed by HAMD-24) and appetite loss.

#### SI

The Positive and Negative Suicidal Ideation Scale (PANSI) was used to assess the degree of SI in subjects over the past two weeks [19]. The scale includes 14 items and uses a 5-point rating system, with higher total scores reflecting more severe SI. The Chinese version of PANSI has been widely used in Chinese adolescents with MDD [20].

#### Daytime sleepiness symptoms

The Chinese version of the Epworth Sleepiness Scale (ESS) was used to assess the degree of daytime sleepiness symptoms in different situations [21]. The scale includes 8 items and uses a 3-point rating system, with higher scores reflecting more severe sleepiness. The ESS scale has good reliability and validity in the Chinese population [22].

#### Insomnia

The Insomnia Severity Index (ISI) includes 7 items to assess the degree of insomnia over the past two weeks [23]. The scale uses a 5-point rating system, with higher scores reflecting more severe insomnia. The ISI scale has been widely used in adolescents with MDD [24].

#### IA

The Chinese version of the Internet Addiction Test (IAT) was used to measure IA [25]. The scale includes 20 items and uses a 5-point rating scale, with higher scores reflecting more severe IA [26]. Previous study has shown that the Chinese version of the IAT was reliable and valid for adolescents [27].

### Statistical analysis

Perform statistical analysis using SPSS23.0. The Kolmogorov-Smirnov test assessed normality of continuous variables. Continuous variables with normal distribution were summarized as mean ± standard deviation (SD), and t-test was used for comparison between groups; measures with non-normal distribution were expressed as M (P_25_, P_75_), and Mann-Whitney U-test was used for comparison between groups. Categorical variables were presented as percentages (%), and the chi-square tests were used for comparison between groups. In multifactorial analyses, stepwise logistic regression analysis was used to identify independent factors associated with appetite loss in adolescents with MDD. Finally, the area under the curve (AUC) was calculated using the receiver operating characteristic (ROC) curve to evaluate the predictive value of each independent factor for appetite loss. Two-sided tests were employed for all statistical analyses, with a significance level set at *p* < 0.05.

## Results

### Comparison of sociodemographic and psychopathological data

In the total sample, the prevalence of appetite loss in adolescents with MDD was 69.2%, with 74.7% in females and 56.1% in males. The results of univariate analysis showed that compared to the non-appetite loss group, patients with appetite loss had a lower BMI (t = 2.759, *p* = 0.006), longer duration of illness (t = 1.372, *p* = 0.020), and higher scores of HAMD (t = -6.387, *p* < 0.001), PANSI (t = -4.178, *p* < 0.001) and ISI (t = -5.021, *p* < 0.001) (Table 1).

### Gender differences between sociodemographic and psychopathological data

Tables 2 and 3 summarized the results of univariate analysis in female and male patients, respectively. In female patients, the scores of HAMD, PANSI and ISI were significantly higher in the appetite loss group than in the non-appetite loss group (*p* < 0.05), while BMI and ESS score was significantly lower in the appetite loss group than in the non-appetite loss group (*p* < 0.05). In male patients, the appetite loss group had higher scores of HAMD, PANSI, ISI and IAT (*p* < 0.05).

**Table 2:** Comparison of sociodemographic and psychopathological data in the appetite loss and non-appetite loss groups in female patients

Variables	Appetite loss (*N* = 192)	Non-appetite loss (*N* = 65)	t/Z/χ^2^	*p*	Cohen’s d
Age (years), mean (SD)	15.01 ± 1.69	15.12 ± 1.60	0.471	0.638	0.068
BMI (kg/m2), mean (SD)	20.20 ± 3.42	21.90 ± 4.16	3.271	0.001*	0.469
Age at onset (years), mean (SD)	13.26 ± 1.86	13.49 ± 2.16	0.832	0.406	0.119
Duration of illness (moths), median (P_25_, P_75_)	18.00 (12.00, 35.00)	12.00 (6.00, 24.00)	1.161 ^a^	0.059	0.072
Antidepressants, n (%)					
None	68 (35.42)	19 (29.23)	0.859	0.651	
SSRIs	109 (56.77)	40 (61.54)			0.058
Others	15 (7.81)	6 (9.23)			
Study pressure					
Small	8 (4.17)	5 (7.69)	5.556	0.062	
Average	67 (34.90)	31 (47.69)			0.147
Large	117 (60.94)	29 (44.62)			
HAMD score, mean (SD)	28.64 ± 7.14	24.25 ± 7.80	-4.182	< 0.001*	0.600
PANSI score, mean (SD)	50.40 ± 11.01	46.14 ± 13.71	-2.268	**0.026**	0.362
ESS score, mean (SD)	9.17 ± 4.66	10.62 ± 4.74	2.157	**0.032**	0.309
ISI score, mean (SD)	13.49 ± 5.52	10.18 ± 6.46	-3.694	< 0.001*	0.573
IAT score, mean (SD)	51.33 ± 15.73	50.85 ± 16.43	-0.211	0.833	0.030

BMI, body mass index; SSRIs, selective serotonin reuptake inhibitors; HAMD, Hamilton depression scale; PANSI, positive and negative suicide ideation inventory scale; ESS, Epworth sleepiness scale; ISI, insomnia severity index scale; IAT, internet addiction test

Bolded *p* value: < 0.05; SD: standard deviation

^a^ Mann-Whitney U-test

Cohen’s d indicates the effect size

* indicated variables with *p* < 0.05 after FDR adjustment

**Table 3:** Comparison of sociodemographic and psychopathological data in the appetite loss and non-appetite loss groups in male patients

Variables	Appetite loss (*N* = 60)	Non-appetite loss (*N* = 47)	t/Z/χ^2^	*p*	Cohen’s d
Age (years), mean (SD)	15.75 ± 1.34	15.79 ± 1.53	0.134	0.894	0.026
BMI (kg/m^2^), mean (SD)	22.72 ± 6.24	22.54 ± 4.54	-0.169	0.866	0.033
Age at onset (years), mean (SD)	15.03 ± 7.69	14.49 ± 1.93	-0.473	0.637	0.092
Duration of illness (moths), median (P_25_, P_75_)	20.00 (6.25, 30.00)	12.00 (4.00, 24.00)	0.710 ^a^	0.463	0.069
Antidepressants, n (%)					
None	27(45.00)	18 (38.30)	3.457	0.178	
SSRIs	31(51.67)	23 (48.94)			0.180
Others	2 (3.34)	6 (12.77)			
Study pressure					
Small	6 (10.00)	1 (2.13)	2.679	0.262	
Average	23 (38.33)	20 (42.55)			0.158
Large	31 (51.67)	26 (55.32)			
HAMD score, mean (SD)	27.92 ± 6.63	21.70 ± 7.79	-4.455	< 0.001*	0.868
PANSI score, mean (SD)	47.10 ± 10.95	41.26 ± 11.06	-2.728	**0.007**	0.531
ESS score, mean (SD)	9.50 ± 4.52	9.26 ± 3.90	-0.295	0.769	0.057
ISI score, mean (SD)	13.45 ± 5.38	10.02 ± 5.58	-3.219	0.002*	0.627
IAT score, mean (SD)	55.40 ± 16.62	49.55 ± 11.98	-2.113	**0.037**	0.396

BMI, body mass index; SSRIs, selective serotonin reuptake inhibitors; HAMD, Hamilton depression scale; PANSI, positive and negative suicide ideation inventory scale; ESS, Epworth sleepiness scale; ISI, insomnia severity index scale; IAT, internet addiction test

Bolded *p* value: < 0.05; SD: standard deviation

^a^ Mann-Whitney U-test

Cohen’s d indicates the effect size

* indicated variables with *p* < 0.05 after FDR adjustment

### Independent factors associated with appetite loss

In the total sample, the results showed that Gender (OR = 0.559, 95% CI = 0.331 ~ 0.944, *p* = 0.029), BMI (OR = 0.925, 95% CI = 0.876 ~ 0.977, *p* = 0.005), HAMD score (OR = 1.080, 95% CI = 1.041 ~ 1.120, *p* < 0.001) and ISI score (OR = 1.073, 95% CI = 1.024 ~ 1.124, *p* = 0.003) were independent correlated of appetite loss in adolescents with MDD. In female patients, BMI (OR = 0.872, 95% CI = 0.806 ~ 0.945, *p* = 0.001), HAMD score (OR = 1.062, 95% CI = 1.016 ~ 1.110, *p* = 0.008) and ISI score (OR = 1.077, 95% CI = 1.017 ~ 1.141, *p* = 0.012) were independently associated with appetite loss. In male patients, only HAMD score (OR = 1.133, 95% CI = 1.062 ~ 1.209, *p* < 0.001) was independently associated with appetite loss (Table 4).

**Table 4:** Independent factors associated with appetite loss by multivariate Stepwise logistic regression analysis

Variables	β	SD	Wald χ^2^	OR	95% CI	*p*
Total sample						
Gender	-0.581	0.267	4.739	0.559	0.331 ~ 0.944	**0.029**
BMI	-0.078	0.028	7.733	0.925	0.876 ~ 0.977	**0.005**
HAMD score	0.077	0.019	17.129	1.080	1.041 ~ 1.120	**< 0.001**
ISI score	0.070	0.024	8.868	1.073	1.024 ~ 1.124	**0.003**
Female						
BMI	-0.136	0.041	11.297	0.872	0.806 ~ 0.945	**0.001**
HAMD score	0.060	0.023	6.977	1.062	1.016 ~ 1.110	**0.008**
ISI score	0.074	0.029	6.341	1.077	1.017 ~ 1.141	**0.012**
Male						
HAMD score	0.125	0.033	14.162	1.133	1.062 ~ 1.209	**< 0.001**

BMI, body mass index; HAMD, Hamilton depression scale; ISI, insomnia severity index scale

Bolded *p* value: < 0.05; SD: standard deviation

OR, odds ratio; CI, confidence interval

### ROC curve analysis of appetite loss

**Fig. 1:**
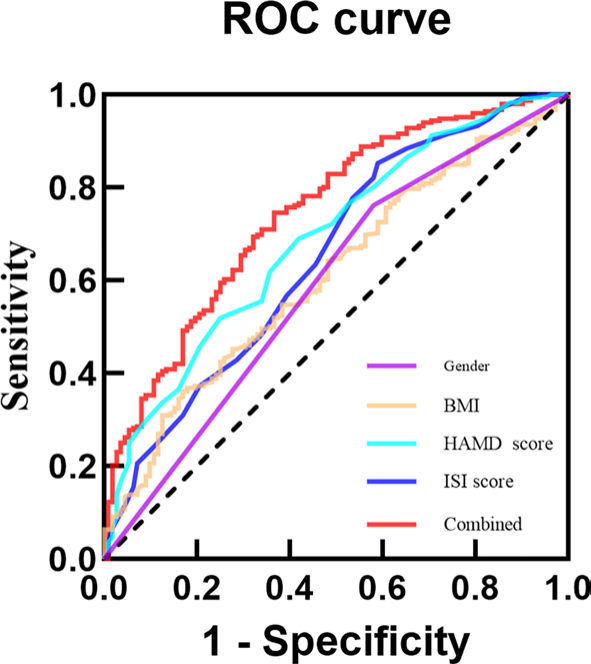
ROC curve analysis of appetite loss in adolescents with MDD in the total sample of Gender, BMI, HAMD score and ISI score

**Fig. 2:**
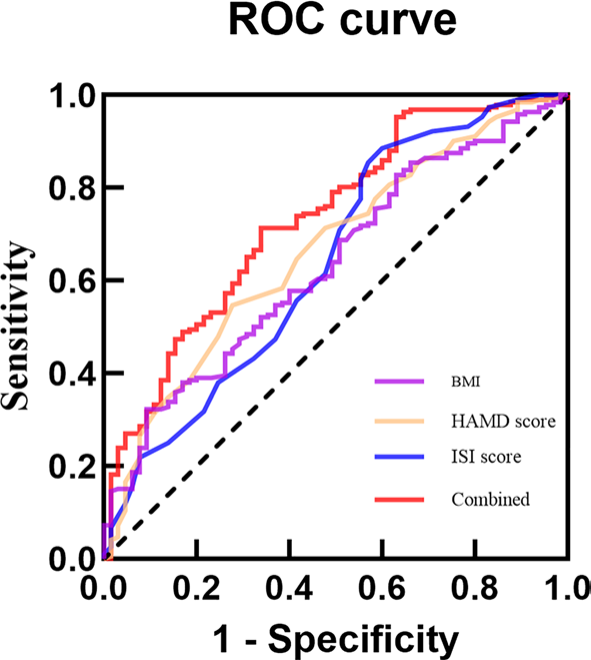
ROC curve analysis of BMI, HAMD score and ISI score on appetite loss in female adolescents with MDD

**Fig. 3:**
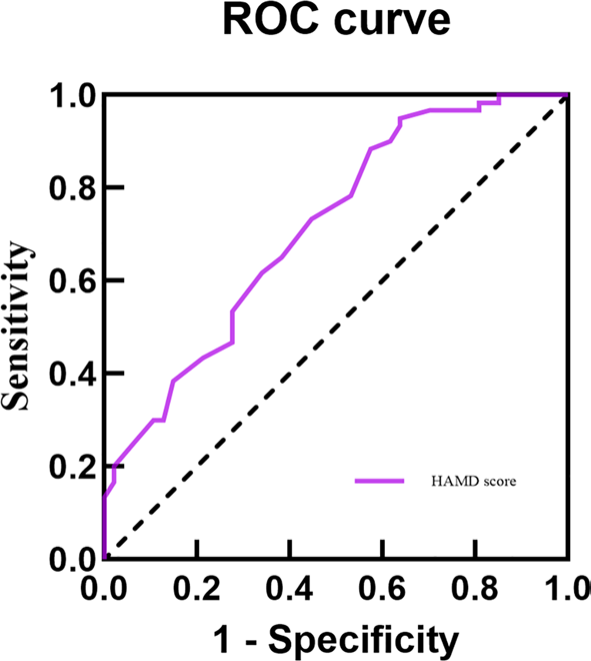
ROC curve analysis of HAMD score on appetite loss in male adolescents with MDD

This study conducted ROC curve analysis to explore the predictive value of independent factors for appetite loss. In the total sample, the results showed that the AUC values for Gender, BMI, HAMD score and ISI score in predicting appetite loss in adolescents with MDD were 0.591, 0.612, 0.689 and 0.652, respectively. Additionally, when these four indicators were combined, the AUC value increased, indicating improved predictive ability for appetite loss (AUC = 0.746, 95% CI = 0.692 ~ 0.800), with enhanced sensitivity and specificity compared to individual indicators (Table 5; Fig. 1). In female patients, the AUC values for BMI, HAMD score and ISI score alone in predicting appetite loss in adolescents with MDD were 0.636, 0.665 and 0.642, respectively. Combining these four indicators y increased the AUC value to 0.732, sensitivity of 71.4% and specificity 66.2% (Table 5; Fig. 2). HAMD score alone predicted appetite loss in male adolescents with MDD with an AUC value of 0.713, sensitivity of 95.0%, and a specificity of 36.2% (Table 5; Fig. 3).

**Table 5:** ROC curve analysis of appetite loss in adolescents with MDD

Variables	AUC	95% CI	Sensitivity(%)	Specificity(%)	*p*
Total sample					
Gender	0.591	0.526 ~ 0.656	0.762	0.420	**0.006**
BMI	0.612	0.551 ~ 0.673	0.361	0.830	**0.001**
HAMD score	0.689	0.631 ~ 0.747	0.691	0.580	**< 0.001**
ISI score	0.652	0.590 ~ 0.715	0.853	0.411	**< 0.001**
Combination	0.746	0.692 ~ 0.800	0.746	0.634	**< 0.001**
Female					
BMI	0.636	0.560 ~ 0.711	0.323	0.908	**0.001**
HAMD score	0.665	0.590 ~ 0.740	0.547	0.723	**< 0.001**
ISI score	0.642	0.560 ~ 0.724	0.885	0.400	**0.001**
Combination	0.732	0.661 ~ 0.811	0.714	0.662	**< 0.001**
Male					
HAMD score	0.713	0.615 ~ 0.846	0.950	0.362	**< 0.001**

BMI, body mass index; HAMD, Hamilton depression scale; ISI, insomnia severity index scale; AUC, area under curve

Bolded *p* value: < 0.05; OR, odds ratio; CI, confidence interval

## Discussion

In this study the results showed that the prevalence of appetite loss in adolescents with MDD was 69.2%, higher than that of adult patients (56.5%) [16]. In terms of gender differences, female patients exhibited a higher incidence rate (74.7%), contrasting with that observed in male patients (56.1%). Currently, the mechanisms underlying gender differences in appetite loss remain incompletely understood. However, relevant study suggested these disparities may relate to gender-specific hormonal variations and differing coping strategies for depressive emotions [28]. Additionally, women often face greater societal pressure regarding body image due to cultural aesthetic standards (e.g.,“thinness equals beauty”) [29]. This dissatisfaction and anxiety about body shape may also be associated with appetite loss [30]. For example, Ferreiro’s research indicated that appetite loss in many adolescent females was linked to dissatisfaction with their body shape [11]. Furthermore, this study found that appetite loss was correlated with lower BMI values, consistent with previous finding in adults [31]. This phenomenon occurring more frequently in females may also be related to their excessive focus on their own body shape. However, future large-scale cohort studies are needed to systematically investigate gender differences in appetite loss in adolescents with MDD, the gender specificity of the association between appetite loss and BMI, and whether such differences persist into adulthood.

In terms of clinical symptoms, appetite loss was associated with the severity of depressive symptoms, previous studies have also yielded consistent results [7, 32]. This association involves multiple potential mechanisms, including psychological and neurobiological mechanisms. First, from a psychological mechanism perspective, depression often manifests as anhedonia, where diminished pleasure from food may trigger avoidance of eating behaviors [33, 34]. Additionally, adolescents with MDD frequently experience emotional dysregulation, which can contribute to the emergence of eating issues [35]. Harrison’s research further revealed that patients with MDD commonly exhibit negative cognitive biases, holding pessimistic views about themselves, their environment, and the future [36]. These negative cognitions may be linked to diminished interest in food; when patients develop the belief that “eating cannot improve mood or bring pleasure” they may experience appetite loss. From a neurobiological perspective, this may also be associated with endocrine dysregulation in depressed individuals, such as abnormally elevated cortisol levels [4, 37].

This study found that appetite loss in patients may be correlated with SI, although the regression analysis did not show statistical significance. This result may be related to the potential influence of antidepressant medication on the association between the two. Studies on adolescent populations indicated that individuals with depression frequently exhibit higher rates of SI [38], and appetite loss can also serve as an early warning indicator for SI and self-harm behaviors [8]. Given the close association between appetite loss and depression, we hypothesize that adolescents with MDD frequently experience emotional distress. This not only heightens the risk of SI but may also diminish interest in food, leading to inadequate nutritional intake. Nutritional deficiencies may further impair physical functioning and psychological resilience, making it harder to cope with life stressors and challenges, thereby intensifying SI. Additionally, interpersonal theories suggested that individuals may perceive themselves as a burden to loved ones, and this sense of burden was closely associated with SI [39]. Adolescents with MDD may harbor similar cognitive patterns, attempting to reduce their perceived burden on the family by eating less. However, current research on the association between SI and appetite loss primarily focuses on individuals with ED. For instance, multiple studies reported relatively higher risks of SI among those with ED [40–42]. Therefore, the specific patterns and underlying mechanisms linking appetite loss to SI in adolescents with MDD require further validation through high-quality research.

Additionally, this study indicated that in adolescents with MDD, female patients exhibit an independent association between appetite loss and insomnia. Previous research has demonstrated that most adolescents with MDD have sleep disorders, usually manifested as insomnia symptoms such as difficulty falling asleep or staying asleep [43]. Current research on the association between appetite loss and insomnia remains predominantly focused on adult and elderly populations. For instance, study in adults has found a strong correlation between appetite loss and insomnia, with reduced sleep duration further amplifying the association between depressive symptoms and insomnia [44]. Studies in the elderly population further revealed that moderate-to-high risk of appetite loss was associated only with insomnia in female seniors [45, 46]. In patients with anorexia nervosa, women typically report more sleep-wake disturbances and difficulty falling asleep [47]. Regarding the mechanisms linking appetite loss and insomnia, research suggested this may involve interactions between orexin, sleep arousal and appetite regulation [48]. For instance, appetite loss may reduce intake of melatonin-rich foods. Melatonin, a hormone secreted by the pituitary gland that plays a role in the sleep-wake cycle, has been reported to enhance sleep efficiency [49]. It is important to note that while previous studies have explored the mechanisms linking sleep and appetite, these mechanisms remain unclear across different age groups and genders. In particular, there was a lack of research on gender-specific mechanisms underlying the association between appetite loss and insomnia in adolescents with MDD. Additionally, this study also found that appetite loss in female adolescent patients with MDD was negatively correlated with sleepiness levels (i.e., sleepiness was positively correlated with increased appetite), although regression analysis did not reveal statistically significant differences. This may be closely related to the small sample size in the female subgroup and insufficient statistical power. Similar findings have been reported in previous studies, such as study on adolescent populations indicated that higher sleepiness levels were associated with stronger food cravings [50]. Similar trends have been observed in adult study, where sleepiness usually accompanies increased appetite [51]. This suggested that the negative association between sleepiness and appetite loss may be consistent across age groups, though further validation is needed. Although our findings indicated that the link between appetite loss and insomnia/sleepiness was more pronounced in female patients, the lack of large adolescent samples with MDD specifically designed to validate this association underscores the need for future studies with expanded sample sizes to confirm both the association and the robustness of gender differences.

Finally, in male adolescents with MDD, IA may be associated with appetite loss, although regression analysis did not show statistical significance. This result may be related to insufficient sample size. A cross-sectional study of adolescents indicated that high-risk male internet users reported skipping meals, appetite loss, and irregular eating patterns more frequently than females [52]. This suggested potential gender differences in the association between internet usage patterns and appetite symptoms, though evidence for its mechanisms in the context of adolescents with MDD remains limited. Varchetta’s research also indicated that males typically engage more enthusiastically than females in internet activities such as gaming and esports [53]. Such behaviors require prolonged focus and sedentary periods, potentially reducing physical activity [54]. This, in turn, may indirectly appetite loss by affecting gastrointestinal function and metabolic rhythms. Based on this, we hypothesized that the association between internet addiction and appetite loss in male adolescents may be linked to sedentary behavior and decreased physical activity. However, current research has not thoroughly examined the specific mechanistic pathways underlying the “internet addiction—sedentary/reduced activity—appetite loss” sequence in adolescents with MDD. Exploration of other potential mechanisms, such as neurobiology, also remains limited. Additionally, mechanistic studies examining gender differences, particularly variations in this association between male and female adolescents with MDD, remain markedly insufficient. Therefore, future large-scale, prospective foundational research is essential to further explore this topic.

However, there were several limitations to this study. First, most patients in this study were taking antidepressants. The cross-sectional design cannot distinguish between drug effects and genuine associations, limiting causal inference. Furthermore, medications were only broadly categorized without specifying particular drugs, dosages, or treatment durations, potentially obscuring key associations or weakening the study’s findings. Future research should document detailed medication information and employ longitudinal designs to further validate the authenticity of these associations. Second, the research measurement tools and indicators were insufficiently refined. Appetite assessment relied solely on a single item from the HAMD, with data primarily based on self-reported information and lacking support from nutritional biomarkers or objective biochemical indicators. Future studies should adopt standardized scales combined with objective biochemical indicators to enhance the accuracy and reliability of results. Third, although this study’s sample originated from eight medical institutions in Anhui Province, both the overall and male subgroup sample sizes were limited. Furthermore, the sole focus on adolescents with MDD and the absence of adult controls may compromise the generalizability of results and hinder the identification of age-related differences. Future studies should expand sample sizes, conduct prospective studies or randomized controlled trials, and include adult controls to refine the design. Fourth, the exploration of gender difference mechanisms was insufficient, with related discussions lacking empirical support. Key indicators such as standardized body image assessment scales and hormone level testing were not incorporated. Subsequent studies should supplement these with appropriate assessment tools and physiological testing, systematically elucidating specific mechanisms through longitudinal data.

## Conclusion

Adolescents with MDD, particularly females, faced a heightened risk of appetite loss. Additionally, appetite loss was significantly associated with multiple clinical symptoms, and these associations showed certain gender differences. Therefore, in clinical management, healthcare providers should pay attention to risk factors associated with anorexia, particularly gender differences. Furthermore, future research should include more high-quality prospective studies to provide deeper insights into the mechanisms underlying appetite loss in this population.

## Data Availability

The data used for this study are available from the corresponding author on reasonable request.
